# Identification of antibodies cross-reactive with woodchuck immune cells and activation of virus-specific and global cytotoxic T cell responses by anti-PD-1 and anti-PD-L1 in experimental chronic hepatitis B and persistent occult hepadnaviral infection

**DOI:** 10.3389/fmicb.2022.1011070

**Published:** 2022-12-06

**Authors:** Christopher P. Corkum, Louisa L. Wiede, Cara L.-A. Ruble, Jiabin Qiu, Patricia M. Mulrooney-Cousins, Meredith A. Steeves, David E. Watson, Tomasz I. Michalak

**Affiliations:** ^1^Molecular Virology and Hepatology Research Group, Division of BioMedical Sciences, Faculty of Medicine, Health Sciences Centre, Memorial University of Newfoundland, St. John’s, NL, Canada; ^2^Lilly Research Laboratories, Elli Lilly and Company, Indianapolis, IN, United States; ^3^Non-Clinical Safety Assessment, Toxicology, Elli Lilly and Company, Lilly Corporate Center, Indianapolis, IN, United States

**Keywords:** woodchuck model of hepatitis B, chronic hepatitis B, occult hepadnaviral infection, immune cell subsets, activation of T cells, programed cell death protein and ligand, Marmota monax

## Abstract

Woodchuck *(Marmota monax)* infected with woodchuck hepatitis virus (WHV) is the most pathogenically compatible naturally occurring model of human hepatitis B virus (HBV) infection, chronic hepatitis B, and HBV-induced hepatocellular carcinoma. This system plays a crucial role in discovery and preclinical evaluation of anti-HBV therapies. Its utilization remains tempered by the relatively narrow range of validated immunologic and molecular tools. We evaluated commercial antibodies against immune cell phenotypic markers and T cell molecules for cross-reactivity with woodchuck antigenic equivalents. The confirmed antibodies against programed cell death protein-1 (PD-1) and its ligand (PD-L1) were examined for *ex vivo* ability to activate WHV-specific, global and bystander cytotoxic T cells (CTLs) in chronic hepatitis and asymptomatic infection persisting after self-resolved acute hepatitis. Examination of 65 antibodies led to identification or confirmation of 23 recognizing woodchuck T, regulatory T, B and natural killer cells, T cell-associated PD-1, PD-L1, CTLA-4 and TIM-3 molecules, CD25 and CD69 markers of T cell activation, and interferon gamma (IFNγ). Antibodies against woodchuck PD-1 and PD-L1 triggered *in vitro* highly individualized WHV-specific and global activation of CTLs in both chronic hepatitis and persistent occult infection. WHV-specific CTLs were more robustly augmented by anti-PD-1 than by anti-PD-L1 in chronic hepatitis, while global IFNγ-positive CTL response was significantly suppressed in chronic hepatitis compared to persistent occult infection. Anti-PD-1 and anti-PD-L1 also occasionally activated CTLs to specificities other than those tested suggesting their potency to trigger side effects. This was particularly apparent when T cells from chronic hepatitis were treated with anti-PD-L1. The current findings indicate that inhibition of the PD-1/PD-L1 pathway could reactivate virus-specific and global T cell responses in both chronic hepatitis and asymptomatic persistent infection. They suggest a mechanism of potential reactivation of clinically silent infection during anti-PD-1/PD-L1 treatment and indicate that this therapy may also subdue occult HBV infection.

## Introduction

Hepatitis B virus (HBV) remains a life-threatening human pathogen that triggers inflammatory liver disease culminating at a high frequency in chronic hepatitis B (CHB), hepatic cirrhosis, and primary hepatocellular carcinoma (HCC; Baumert et al., 2007; Levrero and Zucman-Rossi, 2016). There are an estimated 296 million people with CHB and up to 900 thousand die annually due to liver diseases caused by HBV (World Health Organization, 2022). In addition, up to 2 billion may have a clinically silent but molecularly evident occult HBV infection (OBI) accompanied by a trace replication of pathogenic virus that persists in the absence of circulating HBV surface antigen (HBsAg) when assayed by currently available clinical tests (Raimondo et al., 2019). HBV is also recognized as the most potent human viral carcinogen responsible for most of HCC-related deaths (El-Serag, 2012; Fujiwara et al., 2018; Kim et al., 2018). Its oncogenicity is primarily linked to essentially random integration of viral DNA into hepatocyte genome that modifies expression of genes and compromises stability of the liver genome (Tarocchi et al., 2014; Levrero and Zucman-Rossi, 2016; Tu et al., 2017). It recently became evident that HBV-host genomic fusions are created almost immediately after contact with infectious virus (Chauhan et al., 2017; Tu et al., 2018; Chauhan and Michalak, 2021). There are no treatments eliminating HBV entirely or capable of halting pro-oncogenic process initiated by virus DNA integration. Chronic liver inflammation and accumulation of DNA mutations during cycles of hepatocyte regeneration is also contributor to HBV-related HCC oncogenesis (Liu et al., 2021).

Eastern North American woodchucks (*Marmota monax*) infected with woodchuck hepatitis virus (WHV) represent the closest naturally occurring pathogenic model of HBV infection, CHB and HBV-induced HCC (Summers et al., 1978; Popper et al., 1981; Korba et al., 1987; Michalak, 1998). This infection is particularly valuable for dissecting *in vivo* molecular and immunologic mechanisms underlying HBV infection-associated chronic liver diseases (Michalak, 1998), and for development and pre-clinical evaluation of new therapies, either acting directly on virus or modifying host’s anti-viral immune responses aiming at curtailing persistent infection and its pathological outcomes (Menne and Cote, 2007; Daffis et al., 2021). WHV and HBV are highly compatible regarding genome organization, replication strategy, proteins’ structure and their antigenicity and functions, and immunopathological processes accompanying liver diseases (Galibert et al., 1982; Nassal, 2008; Mason, 2015; Michalak, 2020; Coffin et al., 2021).

A symptomatic infection in WHV-infected woodchucks, like HBV infection in humans, is normally serum WHV surface antigen (WHsAg)-positive (an equivalent of HBsAg) and begins as acute hepatitis (AH) that spontaneously resolves in most adult animals. This self-limited AH (SLAH or SL) is followed by asymptomatic, life-long persistence of virus termed as secondary occult infection (SOI), which equivalence is seropositive-OBI in humans (Michalak et al., 1999; Mak et al., 2020). SOI appears as WHsAg-negative, unless tested after serum or plasma concentration (Coffin et al., 2004), while antibodies to WHV core antigen (anti-WHc; an equivalent of antibodies to HBV core antigen, anti-HBc) are unvaryingly present. The liver shows intermittent minimal to moderate inflammation with periods of normal morphology; nonetheless HCC develops in about 20% of the animals with SOI (Michalak et al., 1999). Circulating WHV DNA occurs at or below 100–200 copies/ml, also called as virus genome equivalents (vge), and molecular indicators of virus replication (i.e., WHV mRNA and covalently closed circular DNA, cccDNA) are detectable in the liver and cells of the immune system (Michalak et al., 1999; Coffin et al., 2004). In the minority (10–15%) of adult animals, like humans infected with HBV during adulthood, AH progresses to chronic hepatitis (CH) that is serum WHsAg and anti-WHc reactive and accompanied by high loads of virus. CH displays features of protracted liver necro-inflammation and advances to HCC in up to 90% of woodchucks (Korba et al., 1989; Michalak, 2020). This rate is much greater than that of about 5% observed in adults with CHB (Korba et al., 1987, 1989; Michalak, 1998). For completeness of the picture, there is yet another form of asymptomatic WHV persistence, primary occult infection (POI; Michalak et al., 2004; Mulrooney-Cousins et al., 2014; Coffin et al., 2021), which has an equivalence in seronegative-OBI in humans (Raimondo et al., 2019). This infection is defined as serum negative for WHsAg, anti-WHc and antibodies to WHsAg (anti-WHs), while WHV DNA is detectable at levels equal to or below 100–200 vge/mL. WHV genome and its replication and integration are identifiable in cells of the immune system and with time also in the liver (Coffin and Michalak, 1999; Mulrooney-Cousins et al., 2014). Liver biochemistry and histology remain normal but HCC develops with a similar frequency as in SOI (Michalak et al., 1999; Mulrooney-Cousins et al., 2014; Michalak, 2020). This form of persistent clinically silent infection was not investigated in the present study.

HBV and WHV are non-cytopathic viruses and liver injury is a consequence of the host’s immune response against the virus and possibly liver autoantigens exposed or modified in the course of hepadnaviral infection (Thimme et al., 2003; Michalak, 2020). It has been demonstrated that cessation of AHB is associated with broad and vigorous HBV-specific reactivity of CD4+ helper T cells and interferon gamma (IFNγ)-positive CD8+ cytotoxic T lymphocytes (CTL; Thimme et al., 2003; Guidotti and Chisari, 2006). Nonetheless, these responses, although resulting in resolution of clinically apparent hepatitis, do not totally eliminate HBV and infection continues as seropositive SOI (Michalak et al., 1994; Rehermann et al., 1996; Raimondo et al., 2019). By contrast, weak and dysfunctional virus-specific T cell response accompanies CHB (Boni et al., 2007; Maini and Schurich, 2010; Park et al., 2016). These T cells are characterized by reduced production of anti-viral cytokines and interleukin-2 (IL-2), attenuated cell cytotoxicity due to engagement of co-inhibitory receptors, including programmed cell death protein 1 (PD-1) and CTL-associated protein-4 (CTLA-4), altered transcriptional and metabolic profiles, and failure in transition to antigen-independent state characterizing memory T cells (Zhang et al., 2009; Schurich et al., 2011, 2013). The blockage of the PD-1/PD ligand 1 (PD-L1) pathway was found to restore effector function of T cells in some patients with CHB and in the woodchuck model of WHV-induced CH (Maier et al., 2007; Fisicaro et al., 2010; Liu et al., 2014; Balsitis et al., 2018). However, reinvigoration of the response greatly varies and ranges from seemingly full restoration to an absence of any measurable effect. The reasons beyond this individualized response are not well recognized. They might be linked to differing expression of PD-1, PD-L1 and other inhibitory molecules on circulating and hepatic lymphocytes, and to a variable involvement of T cell effector and regulatory pathways (Park et al., 2016; Jubel et al., 2020). They could also be related to different clinical profiles of CHB which have been categorized into immune active, immune tolerant and clinically inactive periods (Park et al., 2016; Sung et al., 2019). It is of note that such highly individualized efficacy of PD-1 or PD-L1 blockage is consistent with findings in patients with cancer and infections with other viruses (Nakamoto et al., 2009; Gardiner et al., 2013; Kowanetz et al., 2018; McLane et al., 2019).

Although, WHV-specific T cell responses in woodchucks closely follow those in HBV-infected humans (Hodgson and Michalak, 2001; Menne et al., 2002; Menne and Cote, 2007; Guy et al., 2008; Suresh et al., 2019; Michalak, 2020), their detailed recognition is lagging mainly due to limitation in the range of well characterized antibodies identifying woodchuck immune cells and molecules signifying their functions. The significance of expanding the array of such antibodies is clearly apparent when considering the uniqueness of the model, in which virus infection progresses in the face of an intact immune system which is not manipulated by interspecies immune cell transfer or chemical treatment within host that is naturally susceptible to wild-type, pathogenic virus (Michalak, 2020; Suresh and Menne, 2022).

The current study aimed at evaluation of commercially available antibodies (Abs) for cross-reactivity with phenotypic markers of woodchuck immune cells and selected functionally important molecules on woodchuck T lymphocytes. Abs recognizing woodchuck PD-1 and PD-L1 were subsequently applied to assess functional exhaustion of T cells in experimental CH and SOI. *Ex vivo* activation of WHV-specific and generalized (global) CTL responses was assessed. The results showed that blocking of PD-1 or PD-L1 triggered highly individualized virus-specific and global activation of T cells derived from peripheral blood mononuclear cells (PBMC) of animals with CH as well as animals with SOI continuing after resolution of acute WHV hepatitis. In addition, treatment with anti-PD-1 or anti-PD-L1 in the absence of WHV or mitogenic stimulation occasionally activated bystander T cells with phenotypic characteristics of activated CTLs, suggesting that alternative antigenic specificity triggered these responses.

## Materials and methods

### Animals and categories of WHV infection and hepatitis

This study had two interconnected parts. The first part aimed at the identification of commercially available Abs capable of identifying woodchuck immune cell types and selected molecules reflecting their functional status. In this part, PBMC from healthy woodchucks (*n* = 17), woodchucks infected with WHV with CH (*n* = 3) or with SOI after recovery from SLAH (*n* = 8), as well as from healthy human donors (*n* = 2) were used. For some evaluations, splenocytes isolated from healthy mice were also employed. In the second part, cross-reactive Abs confirmed in the first part of the study were utilized to assess the effect of blockage of PD-1 and PD-L1 on T cell responsiveness to WHV and to global mitogenic stimulation. Animals with experimentally induced CH and SOI continuing after an episode of AH were investigated in this part of the study.

The WHV-naïve status of healthy animals was confirmed by the absence of serological and molecular indicators of WHV infection, including WHsAg and anti-WHc in sera and WHV DNA in sera, PBMC and liver biopsies evaluated by high sensitivity polymerase chain reaction (PCR)-based assays reported before (Michalak et al., 1999; Coffin et al., 2004; Michalak et al., 2004; Mulrooney-Cousins et al., 2014). To induce infection WHV-naïve woodchucks were intravenously (*i.v.*) injected with WHV from an animal chronically infected with WHV7 inoculum (GenBank accession number M18752) at doses equal or greater than 1 × 10^6^ DNase digestion-protected virions. These animal studies were performed at the Woodchuck Viral Hepatitis Research Facility (WHRF) at the Health Science Center, Faculty of Medicine, Memorial University (St. John’s, NL, Canada). The animals with SOI continuing after a self-limiting episode of AH comprised one of the study groups. These animals were serum WHsAg negative, anti-WHc positive and reactive for WHV DNA at ≤ 100–200 vge/ml for 9 to 14 months prior to the study, while low levels of WHV DNA and markers of WHV replication were detectable in liver and PBMC, as reported (Michalak et al., 1999, 2004). Woodchucks with high WHV DNA loads positive for serum WHsAg for longer than 6 months after WHV inoculation were diagnosed as having CH (Michalak et al., 1999). In these animals, robust WHV replication in the liver and the immune system coincided with histologically evident chronic liver inflammation that protracted until the time of collection of PBMC samples predestined for this study. Most of the animals with CH were generated in our WHRF by inoculation of 2-year-old adults, as indicated above. Other adult animals with CH were purchased from the Northeastern Wildlife (Harrison, Idaho, United States). These animals developed CH after injection with WHV7 at dose of ~ 1 × 10^7^ virus genome copies given subcutaneous within 72 h (h) after birth. All animals were housed under environmental and biosafety conditions specifically established for this species in the WHRF at the Health Science Center, Memorial University.

In the study examining the effects of anti-PD-1 and anti-PD-L1 Abs on WHV-specific and generalized CTL response, PBMC from 8 animals with CH, 7 with SOI persisting after an acute episode of WHV hepatitis, and 3 healthy woodchucks were examined. Among animals with CH, 4 developed CH after WHV injection during neonatal period (i.e., woodchucks CH-1 to CH-4; 3 males and one female) which were purchased from the Northeastern Wildlife, while the remaining 4 after WHV inoculation in adulthood (i.e., CH-5–CH-8, three males and one female) which were generated in our WHRF (Table 1).

**Table 1:** Serological markers of WHV infection and liver biochemistry in woodchucks with chronic hepatitis or silent infection persisting after episode of acute hepatitis in samples collected at the time of acquisition of PBMC examined in this study.

Infection category animal number/Sex	Observation period (mo)^a^	Duration of positivity (mo)			Detection at PBMC collection
		WHV DNA^b^	WHsAg^c^	Anti-WHc^d^	WHV DNA^b^ (vge/mL)	WHsAg^c^			Anti-WHc^d^	SDH^e^ (mU/mL)		GGT^f^ (U/L)
*Chronic hepatitis*									

CH-1/M^a^	~31	~31	~31	~31	1.5 x 10^9^	20.7	pos
0	9	CH-2/M^a^	~29	~29	~29	~29	4.4 x 10^8^	21
pos	0	7	CH-3/M^a^	~28	~28	~28	~28	1.3 x 10^6^	7.78	pos	0	0	CH-4/F^a^	~27	~27	~27	~27	3.5 x 10^8^
17.8	pos	0	3	CH-5/M	25	24.5	24	24.5
1.2 x 10^7^	19	pos	0	0	CH-6/F	14	13	13
13	1.8 x 10^8^	19.8		0	0	CH-7/M	12	11.5
11.5	11.5	8.5 x 10^6^	19.5	pos	ns	17	CH-8/M	12
12.5	12.5	12.5	6.0 x 10^7^	20						pos	0	16	*Persistent occult infection*					
				SOI-1/F	23	22	8	22	<10^2^
0.88	pos	ns	5	SOI-2/M	13	12	1.5	12	1.2 x 10^2^
0.73	pos	ns	0	SOI-3/F	26	25	4.5	25
2.8 x 10^2^	0.72	pos	0	0	SOI-4/M	26	25	2
25	<10^2^	0.6	pos	0	0	SOI-5/M	13	12
1	12	<10^2^	0.78	pos	4	0	SOI-6/F	12
11	2	11	<10^2^	0.79	pos	0	0	SOI-7/F
12	11	2	11	<10^2^	0.91	pos	0	0

CH, chronic hepatitis; SOI, secondary occult infection; M, male; F, female; mo, months; SDH, serum sorbitol dehydrogenase; GGT, gamma glutamyl transferase; ns, not suitable for testing.

a The time period in months between inoculation with WHV and collection of PBMC examined. For the marked animals with CH, the estimated time between WHV inoculation in the neonatal period and PBMC collection.

b WHV DNA detected by quantitative PCR; signals below 100 vge/mL were confirmed by semi-quantitative nested PCR and amplicons’ hybridization with a WHV DNA probe, as reported (Michalak et al., 1999; Mulrooney-Cousins et al., 2014; Williams et al., 2018).

c Serum WHsAg evaluated by enzyme-linked immunosorbent assay (ELISA) with sensitivity of 3.25 ng/mL in which values equal to or greater than 2.1 were considered positive and greater values reflected higher loads of circulating antigen (Michalak et al., 1999; Williams et al., 2018).

d Anti-WHc determined by ELISA as reported (Michalak et al., 1999; Coffin et al., 2004).

e SDH, a biochemical measure of liver injury (normal woodchuck values 0-20 IU/L) (Mulrooney-Cousins et al., 2014).

### Isolation of PBMC and splenocytes

Woodchuck and human PBMC were isolated by Ficoll density gradient centrifugation as previously reported (Gujar and Michalak, 2005; Williams et al., 2018). Cell viability assessed *via* trypan blue dye exclusion test using a Countess Automated Cell Counter (Invitrogen, Carlsbad, CA, United States) normally exceeded 95%. In some instances, freshly isolated cells were immediately exposed to test antibodies and analyzed after staining and fixation. Mouse splenic lymphomononuclear cells (splenocytes) were prepared from spleen tissue fragments after a gentle mechanical separation through a fine mesh and density gradient centrifugation, as described before (Michalak et al., 1995, 2000). For some experiments, isolated PBMC and splenocytes were frozen in fetal calf serum (FCS; Invitrogen) containing 10% dimethyl sulfoxide (Thermo Fisher Scientific, Waltham, MA, United States) and stored in liquid nitrogen for further use.

### Peripheral blood mononuclear cells *ex vivo* stimulation

To facilitate identification of some cross-reactive Abs and as T cell stimulation controls, woodchuck PBMC were treated with 25 ng/ml phorbol myristate acetate (PMA; Touraine et al., 1977) and 100 ng/ml ionomycin (both from Sigma-Aldrich, St. Louis, MO, United States) for 18 or 72 h in AIM-V lymphocyte culture medium (Gibco-Invitrogen Corp., Waltham, MA, United States) supplemented with 10% FCS (Gujar et al., 2008). In some experiments, the cells were also cultured with 5 μg/ml of concanavalin A (ConA; Sigma) or 5 μg/ml phytohemagglutinin (PHA; MP Biomedicals, Santa Ana, CA, United States) under similar conditions (Gujar et al., 2008).

To assess WHV-specific T cell responses, PBMC were stimulated for 72 h in duplicates or triplicates with 5 μg/ml of WHV core peptide (WHc97-110) or WHV surface (envelope) peptide (WHs220-234) or with WHx peptide pool encompassing amino acids 1–35 of the WHV X protein (WHx) with each peptide at 5 μg/ml. This pool contained five overlapping peptides which lengths (i.e., the first and the last amino acid positions) and sequences were: peptide 1 (1–15) MAARLCCQLDPTRDV; peptide 2 (6–20) CCQLDPTRDVLLLRP; peptide 3 (11–25) PTRDVLLLRPFSSQS; peptide 4 (15–30) LLLRPFSSQSSGPPF, and peptide 5 (21–35) FSSQSSGPPFPRPSA. For experiments evaluating the activation of T cell responses in the presence of anti-PD-1 or anti-PD-L1, PBMC were exposed for 18 h to the peptide pool containing WHc97-110 and WHs220-234 peptides and WHx peptide pool, which was designated as the total WHV peptide pool.

### Groups of antibodies tested and antibody selection criteria

Due to generally sparse availability of Abs recognizing woodchuck immune cells and molecules signifying their immune function, the focus of the first part of the study was to identify or confirm applicability of commercial Abs detecting such phenotypic markers and molecules. This included testing of Abs against woodchuck CD3 (a marker of T cells and NK T cells), CD8 (a marker of CTLs), FoxP3 (a molecule constitutively expressed by regulatory T cells, T_reg_), NKp46 (CD335; a marker of NK and NK T cells), and CD19 and CD20 (markers of B cells). In addition, cross-reactive Abs recognizing proteins involved in T cell immune function or reflecting their functional exhaustion, including PD-1 (CD279), PD-L1 (CD274), CTLA-4 (CD152), and T cell immunoglobulin and mucin domain containing-3 (TIM-3) were tested. As well, Abs against CD25 and CD95, indicators of T cell activation, were assessed.

In the first step of the Abs selection, woodchuck nucleotide sequences of the molecules of interest were queried from the nucleotide sequence bank of the National Center for Biotechnology Information (NCBI; Bethesda, MD, United States). In parallel, the woodchuck nucleic acid sequences encoding some of these molecules were independently determined during our study (their GenBank accession numbers are listed at the end of “Materials and methods”). The nucleotide sequences were translated into amino acid sequences and compared to those of other species to identify regions of homology. When appropriate, the homologous regions provided a framework to identify potential cross-reactive Abs when the antigenic epitope recognized by a given commercial antibody was reported. In addition, the following factors were considered in selection of Abs: (1) Animal species in which Abs were generated (i.e., Abs produced in rabbits were avoided when possible due to frequent autoimmune cross-reactivity); (2) Immunogen used (i.e., Abs against natural antigens or recombinant full length proteins were favored over Abs generated against peptides; an exception was where the peptide used as an immunogen displayed high homology to the known woodchuck peptide sequence); (3) Species cross-reactivity (i.e., reported reactivity with multiple species was preferred); (4) Clonally (i.e., monoclonal preferred over polyclonal Abs); (5) Fluorochrome-labeled versus unlabeled (i.e., labelled Abs were favored), and (6) Confirmed applications and techniques in which a given antibody was used, and the quality of the data reported. Based on this analysis, 65 Abs purchased from different suppliers were selected for investigations.

### General antibody testing procedure

Testing for cross-reactivity was done by flow cytometry on woodchuck PBMC using human PBMC and mouse splenocytes as positive controls, where appropriate. For primary Abs recognizing extracellular epitopes, 2 × 10^5^–1 × 10^6^ cells per reaction were incubated with test antibody diluted in FACS buffer containing 1 mM ethylenediaminetetraacetic acid (EDTA), 0.1% sodium azide and 2% FCS in phosphate-buffered saline, pH 7.4 (PBS) for 1 h on ice in the dark. If primary Abs were not fluorescently labelled, after washing out the primary Ab, cells were incubated with a fluorophore-conjugated secondary antibody under the same conditions, washed, fixed with 4% paraformaldehyde in PBS for 20 min on ice, and washed again before analysis. For Abs recognizing intracellular epitopes, cells were first fixed, then permeabilized with 0.5% saponin in FACS buffer, and subsequently incubated for 30 min on ice with test antibody.

Given that several antibodies against various antigens were examined in this study, the criteria used to conclude cross-reactivity to woodchuck marker initially varied. Primary testing compared test antibody staining to isotype control staining on woodchuck-derived cells for positivity, but also included positive staining controls against the origin species when available. If potential positive staining was identified, additional testing was done including cells from additional animals and from different stages of WHV-induced disease to demonstrate staining consistency, comparison to known anti-woodchuck antibodies, multiparametric staining to demonstrate positive correlation with other known cell-type specific markers, or comparison of unstimulated and *in vitro* stimulated cells to increase expression. More details on specific staining procedures and controls applied to determine Abs cross-reactivity against particular immune cell markers are provided in Materials and methods, Results and/or in the figure legends.

### Identification of woodchuck T cell subtypes

For staining of woodchuck total T cells, polyclonal Abs of IgG class directed against human CD3 epsilon (DAKO Denmark A/S, Glastrup, Denmark) were utilized. These Abs visualize T cells in an indirect reaction after incubation with flourochrome-conjugated anti-rabbit Abs and they have been found to satisfactorily identify woodchuck CD3+ T cells by flow cytometry (Menne et al., 1997), and this was also confirmed in our study. We employed these Abs for multicolor staining over the newly identified anti-CD3 Abs (see Table 2) because of their well-known compensation parameters and consistency in specific staining. Woodchuck CD4+ T cells were detected with fluorochrome-labelled monoclonal antibody (mAb) against human CD4 (clone L200; BD BioScience, Franklin Lakes, NJ, United States) which cross-reactivity with woodchuck T lymphocytes was established before (Balsitis et al., 2018) and ascertained in the course of this study (Table 2). Since identification of woodchuck CD8+ T cells remained unsatisfactory after testing of several (*n* = 11) Abs from different suppliers, particularly when they were used in conjunction with other cross-reactive Abs for multicolor staining, an indirect approach to detect activated CTL previously developed in this laboratory was used (Mulrooney-Cousins and Michalak, unpublished; Michalak, 2020). This method is based on the detection of interferon gamma (IFNγ)-positive cells within CD4-negative cells gated from the total CD3-positive T cell population, i.e., CD3+/CD4-/IFNγ+ T cells (Supplementary Figure 1). The protocol utilizes contrastingly labelled woodchuck cross-reactive anti-CD4 clone L200 mAb (BD BioScience) and anti-bovine IFNγ mAb (Mabtech Inc., Cincinnati, OH, United States) which has been identified in our laboratory as recognizing woodchuck IFNγ (Jenkins, Mulrooney-Cousins, and Michalak, unpublished; Table 2), and enumeration of CD3+/CD4–/IFNɣ+ T cells. Based on the phenotypic criteria, these cells were considered to be activated CTLs. Occasionally, staining for CD3 and IFNɣ was applied to identify these cells since the cumulative data showed that the numbers of gated CD3+/CD4–/IFNɣ+ and CD3+/IFNɣ+ lymphocytes were essentially identical when PBMC from woodchucks with different forms of WHV infection and stages of hepatitis were examined.

**Table 2:** Antibodies cross-reactive with woodchuck immune cell phenotypic markers and functionally important T cell molecules identified or confirmed in the course of this study.

Immunogen	Antibody type	Host species	Immunogen species	Clone	Antibody isoptye	Supplier
CD3	M	mouse	rat	G4.18	IgG3	BD Biosciences
	M	mouse	human	APA1/1	IgG1	BioLegend
	M	rat	human	CD3-12	IgG1	AbD Serotec
	P	rabbit	human	N/A	IgG	DAKO
CD4	M	mouse	human	L200	IgG1	BD Biosciences
FoxP3	M	mouse	human	259D/C7	IgG1	BD Biosciences
	M	rat	mouse	FJK-16s	IgG2a	eBioscience
NKp46	P	goat	mouse	N/A	IgG	R&D Systems
	M	mouse	human	195314	IgG2b	R&D Systems
	M	mouse	human	MM0491-8F24	IgG2b	Abcam
CD19	P	rabbit	human	N/A	IgG	Cell Signaling
CD20	P	goat	mouse	N/A	IgG	Santa Cruz*
PD-1	M	hamster	mouse	J43	IgG	eBioscience
	M	mouse	human	EH12.2H7	IgG1	BioLegend
PD-L1	M	rat	mouse	MIH5	IgG2a	eBioscience
	M	mouse	human	29E.2A3	IgG2b	BioLegend
CTLA-4	M	mouse	human	F-8	IgG1	Santa Cruz
	M	mouse	human	L3D10	IgG1	BioLegend
	P	goat	mouse	N/A	IgG	Santa Cruz*
TIM3	M	rat	human	MM0936-14S23	IgG1	Abcam
CD25	M	mouse	human	IL2R.1	IgG1	Thermo Fisher
CD69	M	hamster	mouse	H1.2F3	IgG	BioLegend
IFNγ	M	mouse	bovine	N/A	IgG	Mabtech

M, monoclonal antibody; P, polyclonal antibodies; N/A, not available or not applicable; * discontinued.

### Validation of woodchuck cross-reactive anti-PD-1 and anti-PD-L1 antibodies

To confirm specificity of commercially acquired anti-PD-1 mAb, clone J43 (eBioscience, San Diego, CA, United States) and clone EH12.2H7 (BioLegend, San Diego, CA, United States), as well as anti-PD-L1 mAb, clone MIH5 (eBioscience) and clone 29E.2A3 (BioLegend), HEK293T cells transiently transfected with recombinant woodchuck PD-1 (wcPD-1) and woodchuck PD-L1 (wcPD-L1) were examined as additional test targets. For this purpose, sequences encoding wcPD-1 and wcPD-L1 were determined, expression vectors constructed using pCDNA 3.1(+), and HEK293T cells transfected using PolyJet DNA transfection reagent (SignaGen Laboratories LLC, Gaithersburg, MD, United States). Surface and intracellular expression of the transfected protein was determined by flow cytometry and compared to non-transfected cells and an isotype control.

### Measurement of the effect of anti-PD-1 or anti-PD-L1 on activation of WHV-specific and global IFNγ-positive cytotoxic T cells

Woodchuck PBMC were cultured in 48-well flat-bottomed plates (Thermo Fisher Scientific, Waltham, MA, United States) at a density of 1 × 10^6^ cells/well in complete AIM-V medium with 10% FCS. The cells were stimulated with the total WHV peptide pool for 18 h in duplicate or triplicate in the presence of anti-PD-1 clone J43 (50 μg/ml; eBioScience) or anti-PD-L1 clone MIH5 (50 μg/ml; eBioScience). Since the assay is based on detection on intracellular IFNγ, which is readily released from activated T cells, treatment of the cells with 4 μM monensin for final 7 h of the 18-h stimulation was applied to limit secretion of the cytokine. In addition, cells cultured under the same conditions in the presence of anti-PD-1 or anti-PD-L1 and PMA/ionomycin served as stimulation controls and to assess the cells’ global activation. Also, cells treated with anti-PD-1 or anti-PD-L1 in the absence of WHV peptides or PMA/ionomycin were used to identify whether Abs alone can activate bystander T cells. In all experiments testing the blocking effect of anti-PD-1 or anti-PD-L1 mAbs, treatment with the matched immunoglobulin (Ig) isotype (i.e., mouse IgG1) served as negative control. The cells were harvested, washed twice with FACS buffer, and incubated with a live/dead cell marker using the ZombieAqua Fixable Viability Kit (BioLegend) for 15 min at ambient temperature. The surface of cells was stained with PE-conjugated CD4 mAb (clone L200; BD BioScience) for 45 min on ice, fixed and then permeabilized with Foxp3/Transcription Factor Staining Buffer Set (eBioScience) according to the manufacturer’s protocol. Subsequently, the cells were incubated with unconjugated anti-CD3 antibody (DAKO) for 30 min on ice before incubating simultaneously with AF647-conjugated donkey anti-rabbit IgG (Jackson Immunoresearch Laboratories, Bar Harbor, ME, United States) and FITC-conjugated anti-IFNγ (Mabtech) for 45 min. The cells were washed twice with either FACS buffer or Perm Wash Buffer (eBioScience) after each antibody staining. Experiments included incubations with matched immunoglobulin controls including Armenian hamster IgG isotype control (clone eBio299Arm; eBioScience) or rat IgG2a *kappa* isotype control (clone eBR2a; eBioScience).

### Acquisition and analysis of flow cytometry data

Data were acquired using a MoFlo Astrios EQ or a Cytoflex flow cytometer (both from Beckman Coulter, Mississauga, Ontario, Canada) and their analysis carried out with FlowJo v10 software (Beckman Coulter). The gating strategy typically involved doublet cell signal exclusion by forward scatter (FSC)-width against FSC-height (~95% singlets) followed by gating cells on FSC-height against side scatter (SSC)-height for debris exclusion (90–95% PBMC; Supplementary Figure 1). Cells were then plotted on FSC-area against Zombie Aqua stained-area to differentiate between live and dead cells (95–99% viable cells) before gating on CD3+ (50–75% cells) and/or CD4+ T cells (26–30%). In the case of activated CTL population, cells were gated as CD4-/IFNɣ+ from CD3+ cells, i.e., CD3+/CD4-/IFNγ+ T cells. The percentage of change in number of CTLs after stimulation with individual WHV peptides, the total WHV peptide pool or PMA/ionomycin was calculated using formula: (number of cells after stimulation) – (number of cells unstimulated) / (number of cells unstimulated) x 100. The percent of change in number of CTLs following treatment with anti-PD-1 or anti-PD-L1 mAbs and stimulation or not with the total WHV peptide pool or PMA/ionomycin was calculated based on formula: (number of cells after treatment with mAb and stimulation with WHV peptide pool or PMA/ionomycin) – (number of cells after treatment with matched Ig isotype and stimulation with WHV peptide pool or PMA/ionomycin)/(number of cells after treatment with matched Ig isotype and stimulation with WHV peptide pool or PMA/ionomycin) × 100.

### Statistical analysis

Results were analyzed by unpaired, nonparametric Mann Whitney test using GraphPad Prism Software (Graph Pad Software Inc., San Diego, CA, United States). Differences between animal groups and treatments were considered to be significant when two-tailed exact *p*-value from analysis of mean cell numbers was equal or lower than 0.05.

### Accession numbers of woodchuck sequences identified in this study

Woodchuck (wc) gene sequences identified in the course of this study and their GenBank accession numbers are as follow: wcCD8a (ON921723), wcCD8b (ON921724), wcCD19 (ON921725), wcCD274 also known as PD-L1 (ON921726), wcFoxP3 (ON921727), wcHAVCR2 also known as TIM3 (ON921728), wcIL-2 (ON921729); wcNCR1 as known as CD335 (ON921730), wcPDCD1 also known as PD-1 and CD279 (ON921731).

## Results

### Cross-reactive antibodies recognizing woodchuck immune cell subtypes

**Figure 1:**
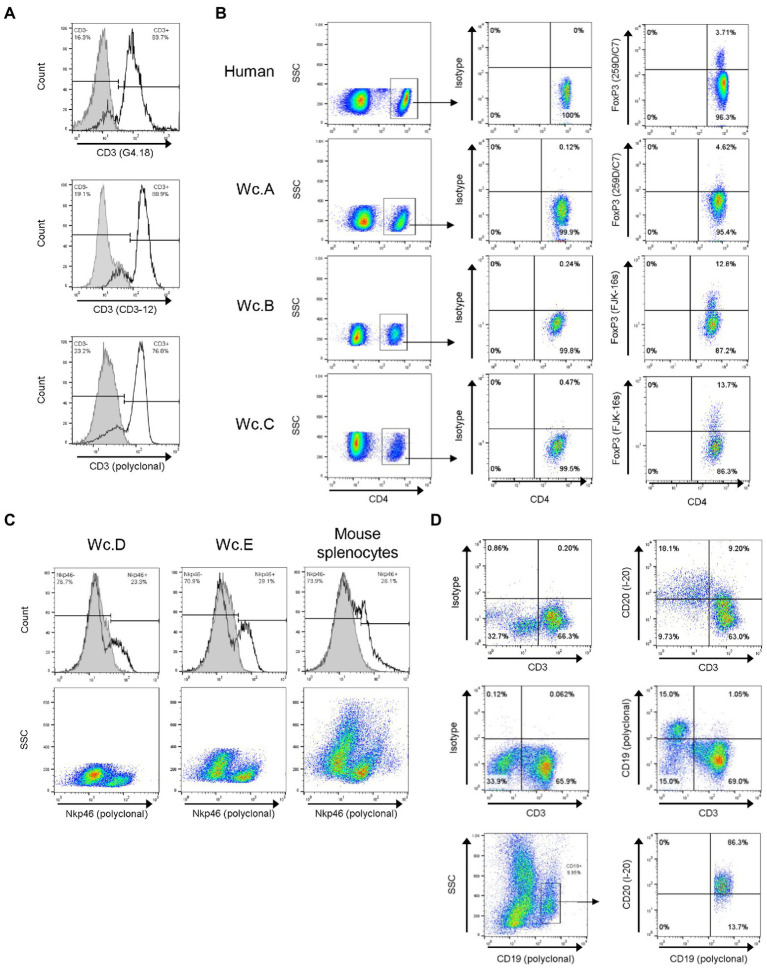
Identification of cross-reactive antibodies against woodchuck T, NK and B cells. **(A)** Cross-reactivity of mouse anti-rat CD3 (clone G4.18), rat anti-human CD3 (clone CD3-12), and rabbit anti-human CD3 epsilon (polyclonal), which served as a positive control, with healthy woodchuck PBMC (unfilled histograms). Filled histograms represent isotype controls. Horizontal line segments demark cells negative or positive with a given antibody, while respective cell percentages are shown in left or right corners of each panel. **(B)** Examples of cross-reactivity of mouse anti-human FoxP3 (clone 259D/C7) and rat anti-mouse FoxP3 (clone FJK-16 s) antibodies with woodchuck T cells. PBMC from three animals (Wc.A, Wc.B, and Wc.C) stained with anti-CD4 and anti-FoxP3 (right column) or with anti-CD4 and isotype controls (middle column) are shown. FoxP3 expression was examined on gated CD4+ T lymphocytes, as shown in left column. PBMC from five healthy woodchucks were analyzed in total with each anti-FoxP3 antibody. The percentage of CD4+ T cells expressing FoxP3 ranged from 2.5 to 4.5% for 259D/C7 clone and from 1.0 to 13.7% for FJK-16 s clone for all five animals. Staining of human PBMC is shown as a positive control in top row. **(C)** Cross-reactivity of goat anti-mouse Nkp46 with healthy woodchuck PBMC. PBMC from two woodchucks (Wc.D and Wc.E) were stained with anti-Nkp46 (unfilled histograms) or with an antibody isotype control (filled histograms). Isolated mouse splenocytes served as the staining positive control (panels on left). Dot plots of the histograms are presented in bottom row. See the legend for panel A for other details. **(D)** Cross-reactivity of goat anti-mouse CD20 and rabbit anti-human CD19 with healthy woodchuck PBMC. The cells were stained with anti-CD3 and anti-CD19 or with anti-CD3 and anti-CD20, and with anti-CD3 and matched isotype controls. Lymphocyte population was identified as CD3+ cells after FSC vs. SSC gating. CD20+ cells (top row) and CD19+ cells (middle row) are shown in top left quadrants. Co-expression of CD19 and CD20 after gating on CD19+ cells is presented in bottom row. More than 86% of CD19+ woodchuck B cells expressed CD20.

By applying flow cytometry, 4 from 6 Abs against CD3 selected for investigation were identified as cross-reactive with woodchuck lymphocytes (Table 2). Among the reactive Abs, 3 were mAbs of which two were generated in mice against either rat CD3 (clone G4.18; BD BioScience; Figure 1A; clone G4.18; Lu et al., 2005; Fisicaro et al., 2010) or human CD3 (clone APA1/1; BioLegend), and the third mAb was produced in rat against human CD3 epsilon (clone CD3-12; AbD Serotec, Oxford, United Kingdom; Figure 1A; clone CD3-12). The fourth was polyclonal Abs against human CD3 epsilon (DAKO; Figure 1A; polyclonal), which were previously applied to identify woodchuck CD3 T cells (Menne et al., 1997) and were used as a positive control in the current study. Except for clone APA1/1 which stained low numbers of woodchuck cells (data not shown), clones G4.18 and CD3-12 and polyclonal anti-human CD3 epsilon Ab serving as a control identified comparable percentages of T cells in the same animals, as exemplified for PBMC from a heathy woodchuck in Figure 1A.

An extensive search was undertaken in an attempt to identify commercial Abs recognizing woodchuck CD8 by flow cytometry. Eleven Abs were tested, the majority of which (*n* = 10) were mAbs produced against CD8 alpha molecule of human or other species. However, none of them were capable of specific identification of woodchuck CD8+ T cells when examined under stringent flow cytometry control conditions (data not shown). It was found that differentiation between viable and dead cells was of paramount importance to reliably identify Abs reactive with woodchuck CD8.

Five Abs against Foxp3, a molecule constitutively displayed by T_reg_ cells, were selected for testing. Mouse anti-human Foxp3 (clone 259D/C7) from BD Biosciences (Figure 1B; clone 259D/C7) and rat anti-mouse Foxp3 (clone FJK-16 s) from eBioscience (Figure 1B; clone FJK-16 s) were found to recognize woodchuck lymphocytes (Table 2).

Twelve mAbs or polyclonal Abs against CD335 (NKp46) from 5 different suppliers were examined. Three of them showed promising results, although also exhibited relatively high background staining. One of them, goat anti-mouse NKp46 from R&D Systems (Minneapolis, MN, United States), gave the most satisfying results and a consistent staining of lymphocytes from different healthy woodchucks (Figure 1C; Table 2). Staining of isolated mouse splenocytes with the same Ab served as a positive control (Figure 1C).

Among 6 Abs against CD19 (*n* = 4) or CD20 (*n* = 2), molecules which are specifically expressed on B lymphocytes, two demonstrated cross-reactivity with woodchuck B cells and both were polyclonal Abs (Table 2). One was produced against human CD19 (Cell Signaling, Danvers, MA, United States) and another against mouse CD20 (Santa Cruz). The woodchuck B cell specificity of the Abs was ascertained by CD3 negativity of the positive cells (Figure 1D) and by the fact that more than 85% of cells from the same animals were recognized by both anti-CD19 and anti-CD20 (Figure 1D). However, the manufacturer has since discontinued production of the anti-CD20 Abs found to be cross-reactive with woodchuck B cells in this study. It should be mentioned that cross-reactivity of the above polyclonal Abs, as well as clinically applicable humanized mouse mAb against human anti-CD20 (Rituximab; Genentech, Forest City, CA, USA), with woodchuck B cells was uncovered in our previous study (Michalak et al., 2019; Mulrooney-Cousins and Michalak, unpublished data/manuscript in preparation).

### Antibodies against functionally important molecules on woodchuck T cells

**Figure 2:**
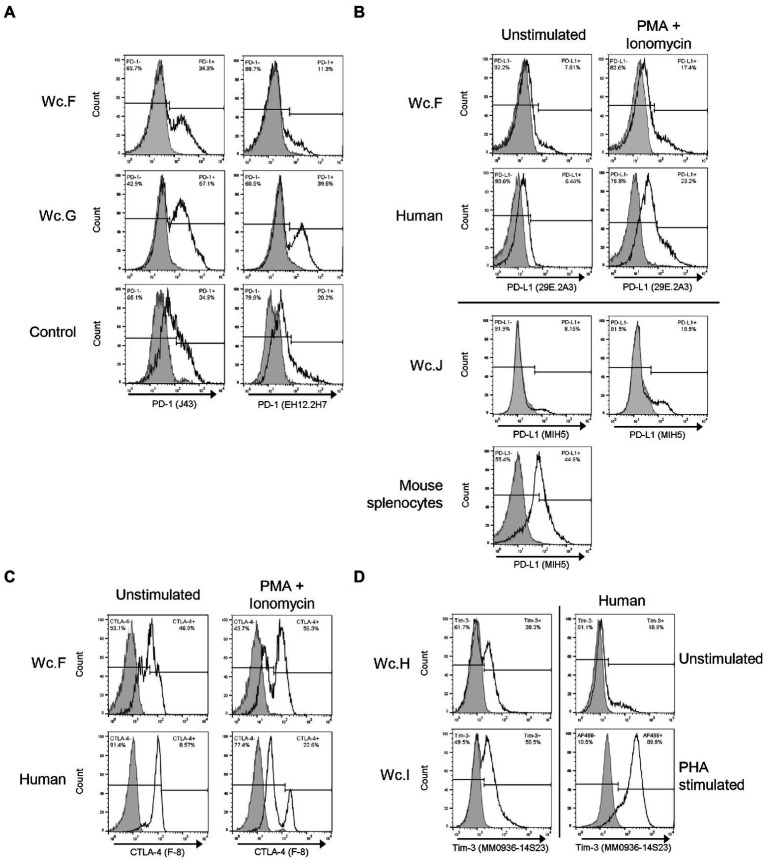
Identification of cross-reactive antibodies recognizing markers of immune exhaustion on woodchuck T cells. **(A)** Cross-reactivity of hamster anti-mouse PD-1 (clone J43) and mouse anti-human PD-1 (clone EH12.2H7) with woodchuck PBMC (unfilled histograms). PBMC from two woodchucks (Wc.F and Wc.G) with serum WHsAg-positive chronic hepatitis were examined. PD-1 expression on mouse splenocytes and human PBMC stimulated with ConA are shown, respectively, for comparison (Control). Filled histograms represent isotype controls. Horizontal line segments demark negative or positive cells, while respective cell percentages are shown in left and right corners of each panel. **(B)** Cross-reactivity of rat anti-mouse PD-L1 (clone MIH5) and rat anti-mouse PD-L1 (clone 29E.2A3) with PBMC from two woodchucks (Wc.F and Wc.J) with serum WHsAg-reactive chronic WHV infection (unfilled histograms). PBMC were stimulated with PMA/ionomycin as described in Materials and methods. PD-L1 expression on similarly stimulated human PBMC and mouse splenocytes are shown, respectively, as controls. Filled histograms represent isotype controls. See the legend for panel A for more details. **(C)** Cross-reactivity of mouse anti-CTLA-4 (clone F-8) with PBMC from a woodchuck (Wc.F) with serum WHsAg-positive chronic WHV hepatitis (unfilled histograms). PBMC were stimulated with PMA/ionomycin. Human PBMC are shown as a positive control. Filled histograms represent staining with isotype controls. See the legend for panel A for additional details. **(D)** Cross-reactivity of rat anti-human TIM-3 (clone MM0936-14S23) with woodchuck PBMC (unfilled histograms). PBMC from two woodchucks (Wc.H and Wc.I) with chronic WHV hepatitis are presented. TIM-3 expression on human PBMC stimulated with PHA is shown as a positive control. Filled histograms represent isotype controls. For other details, see the legend for panel **A**.

Three mAbs and one polyclonal Abs against mouse or human PD-1 were examined for reactivity with woodchuck cells. Two of the Abs which were manufactured by eBioscience and both were previously reported to be cross-reactive with woodchuck PD-1, i.e., Armenian hamster mAb to mouse PD-1 (clone J43; Liu et al., 2014) and mouse mAb against human PD-1 (clone J116; Zhang et al., 2011). However, in our hands, clone J43 was only found to be cross-reactive (Figure 2A; clone J43; Table 2). Among two other Abs tested, mouse mAb to human PD-1 (clone EH12.2H7; BioLegend), which also recognized PD-1 of marmoset, rhesus, squirrel monkey and cynomologus, was cross-reactive with woodchuck PD-1 when tested on ConA-or PMA/ionomycin-stimulated or unstimulated PBMC from healthy and WHV-infected animals (Figure 2A; clone EH12.2H7; Table 2). Despite the fact that J43 and EH12.2H7 were evidently cross-reactive when using stimulated woodchuck PBMC, these mAbs did not recognize HEK293T cells transfected with wcPD-1, either by cell surface or intracellular staining (not shown). A reason behind this remains uncertain but could be related to insufficient transfection efficiency and/or low expression of wcPD-1 protein. However, increasing the amounts of the vector carrying wcPD-1-encoded sequence for transfection of HEK293T cells did not increase protein detection.

Regarding PD-L1, 6 different mAbs generated either in mouse or rat were selected for examination. One mAb, rat anti-mouse PD-L1 clone MIH5 from eBioscience, was previously identified as woodchuck cross-reactive (Zhang et al., 2011), and this was also confirmed in our study (Figure 2B; clone MIH5; Table 2). Another mouse mAb found to be woodchuck reactive was clone 29E.2A3 from BioLegend produced by immunization with human PD-L1 (Figure 2B; clone 29E.2A3; Table 2). This mAb also recognized PD-L1 of chimp, marmoset, rhesus and squirrel monkey, as reported by the manufacturer. In contrast to woodchuck cross-reactive anti-PD-1 Abs, both anti-PD-L1 MIH5 and 29E.2A3 clones efficiently recognized HEK293T cells transfected wcPD-L1 (not shown).

Five Abs directed against human or mouse CTLA-4 were tested using woodchuck PBMC stimulated *ex vivo* with PMA/ionomycin, ConA or PHA. Two of the mAbs were identified as cross-reactive and both were raised against human CTLA-4. They were clone F-8 from Santa Cruz (Figure 2C; clone F-8) and clone L3D10 from BioLegend which stained a lower cell number than F-8 clone (Table 2). F-8 also recognized CTLA-4 of rat, bovine, porcine, equine and canine, as reported by the supplier. It should be mentioned that goat anti-mouse CTLA-4 (Santa Cruz), which were reported previously as woodchuck cross-reactive (Yang et al., 2003; Lu et al., 2005), also recognized woodchuck CTLA-4 in our hands, but these Abs are no longer available.

Five anti-TIM-3 Abs were tested, among which 3 were mAbs and 2 polyclonal Abs. Flow cytometry examinations using PBMC from healthy and WHV-infected animals, which were *ex vivo* stimulated with ConA, PHA or PMA/ionomycin, were performed. Rat mAb against human TIM-3 (clone MM0936-14S23; Abcam, Cambridge, MA, United States) was identified as the only one giving consistent staining of woodchuck lymphocytes (Figure 2D; Table 2).

### Antibodies identifying markers of woodchuck T cell activation

**Figure 3:**
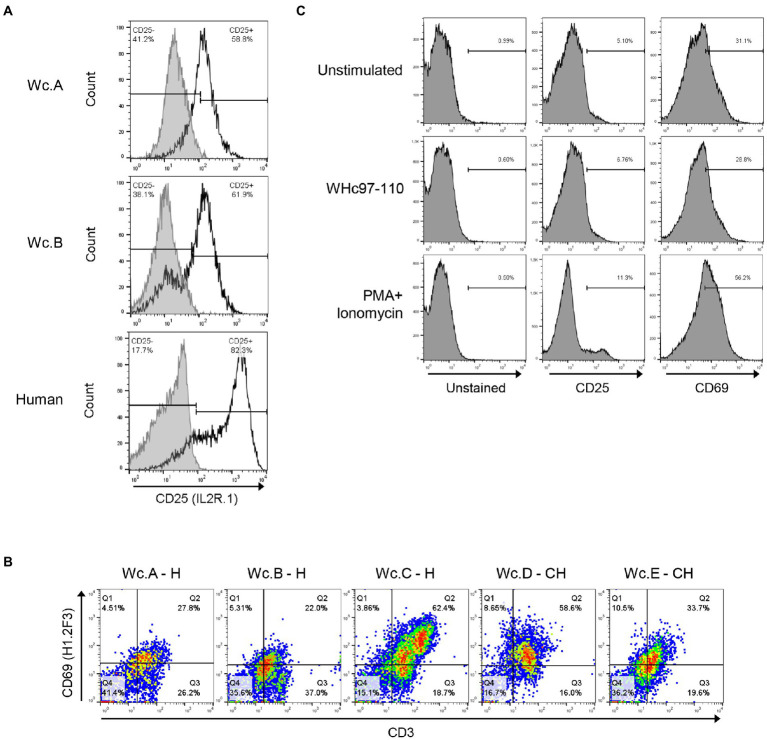
Identification of antibodies cross-reactive with woodchuck markers of T cell activation. **(A)** Cross-reactivity of mouse anti-human CD25 (clone IL2R.1) with woodchuck PBMC (unfilled histograms). PBMC from two healthy animals (Wc.A and Wc.B) were stimulated with ConA as described in Materials and methods. Similarly stimulated human PBMC are shown as a positive control. Cells stained with matched isotype controls are shown as grey-filled histograms. Horizontal line segments demark cells negative or positive with a given antibody and respective cell percentages are presented in left and right corners of each panel. **(B)** Flow plots of PBMC obtained from three healthy (H) and two woodchucks with chronic WHV hepatitis (CH) after double staining with anti-CD3 and anti-CD69 (clone H1.2F3) antibody. Percentages of CD3+/CD69+ cells are shown in the top right corners of each panel. **(C)** Surface expression of CD25 and CD69 on cells from a woodchuck with chronic WHV hepatitis following *in vitro* stimulation of PBMC with WHc97-110 peptide or PMA/ionomycin, and staining with antibodies against CD25 (clone IL2R.1) or CD69 (clone H1.2F3). Horizontal line segments demark positive cells and their percentages are shown above the lines.

Among 5 anti-CD25 Abs tested by employing ConA-or PHA-stimulated woodchuck and human PBMC as targets, two were found promising and were examined further. One of them, mouse mAb anti-human CD25 (clone IL2R.1; Thermo Fisher) showed consistent well-defined staining of T cells from several woodchucks (Figure 3A; clone IL2R.1; Table 2). One of the other mAbs (clone M-A251; BioLegend) did not stain woodchuck cells by flow cytometry in our hands, although otherwise has been reported (Crettaz et al., 2009; Otano et al., 2012).

Regarding anti-CD69 Abs, two clones FN50 and H1.2F3, both from BioLegend, were selected for testing based on the previous studies using cells from other species. Clone FN50 did not show reactivity with woodchuck PBMC stimulated with ConA or PMA/ionomycin, while clone H1.2F3 produced in hamster against mouse CD69 stained between 22 and 62% of the stimulated lymphocytes from either healthy or WHV-infected animals with CH (Figure 3B; clone H1.2F3; Table 2). In this regard, when PBMC from a woodchuck with CH were stimulated with WHV peptide WHc97-110 or PMA/ionomycin and then stained with clone IL2R.1 (anti-CD25) or clone H1.2F3 (anti-CD69), the number of CD25-positive cells increased by about 2% after exposure to the WHV peptide, while that of CD69 reactive cells remained unchanged comparing to unstimulated controls. Further, stimulation with PMA/ionomycin augmented more CD69 (increase by ~ 25%) than CD25 (increase by ~ 6%) on the same lymphocytes (Figure 3C). Testing T cell activation in two other woodchucks with CH showed comparable results, i.e., CD25 expression tended to increase after stimulation with WHc97-110 peptide more than that of CD69, while the reverse was seen after stimulation with PMA/ionomycin (not shown). The results might suggest that CD25 and CD69 could be differentially upregulated on T cells in response to different stimuli.

### Activation of woodchuck CD3+/CD4-/IFNɣ+ T cells in response to WHV and mitogen stimulation

Since functionality of the assay based on identification of CD3+/CD4-/IFNɣ+ T lymphocytes in detecting woodchuck activated CTL cells was not reported in detail before, the initial experiments were performed to confirm the assay applicability. In the first step, PBMC from 3 healthy and 3 WHV-infected woodchucks were stimulated with ConA or PMA/ionomycin followed by staining for CD3 and IFNɣ. Flow cytometry results showed that all animals exhibited an increase in numbers of IFNɣ+ T cells in response to stimuli, although to variable levels, as illustrated for selected animals in Supplementary Figures 2A,B.

**Figure 4:**
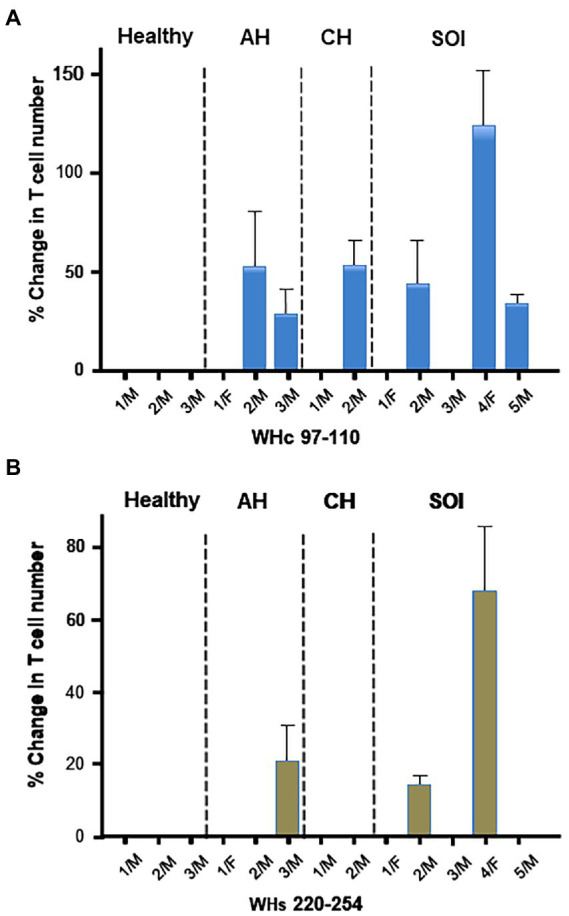
Activation of WHV-specific cytotoxic T cells with the CD3+/CD4-/IFNγ+ phenotype after stimulation of PBMC from WHV-infected and healthy woodchucks with WHV peptides. Percentage changes in numbers of CD3+/CD4-/IFNγ+ T lymphocytes were determined after cell stimulation with **(A)** WHc97-110 peptide or **(B)** WHs220-254 peptide. The cells treated under same conditions in the absence of WHV peptides served as unstimulated controls. PBMC were obtained from healthy animals, woodchucks with acute hepatitis (AH), chronic hepatitis (CH) and secondary occult infection (SOI). For calculation of the cell number percentage change, see Materials and methods. The values from a given animal represent means with value ranges from up to 6 evaluations of at least two PBMC samples.

CD3+/CD4-/IFNɣ+ T cells were also enumerated after stimulation with WHc97-110 or WHs220-234 peptide of PBMC from animals with AH, CH or SOI and from healthy woodchucks. Increases in IFNɣ+ T cell numbers were highly variable among WHV-infected animals (Figure 4). The cells from some animals became activated in response to both WHV peptides, i.e., 1/3 animals with AH and 2/5 with SOI, while others to WHc97-110 peptide only, i.e., 1/3 with AH, 1/2 with CH, and 1/5 with SOI (Figure 4). Overall, cells from 6 of 10 animals infected with WHV demonstrated virus-specific activation, while the remaining animals did not show a measurable response when compared to unstimulated controls. WHV-specific reactivity was not detected in PBMC from healthy woodchucks. Overall, the results implied that enumeration of CD3+/CD4-/IFNɣ+ T lymphocytes is a valuable approach when assessing WHV-specific and global T cell responses in the woodchuck model.

### Blocking of PD-1 and PD-L1 induces highly individualized WHV-specific and generalized cytotoxic T cell activation in chronic hepatitis and occult infection persisting after resolution of acute hepatitis

**Figure 5:**
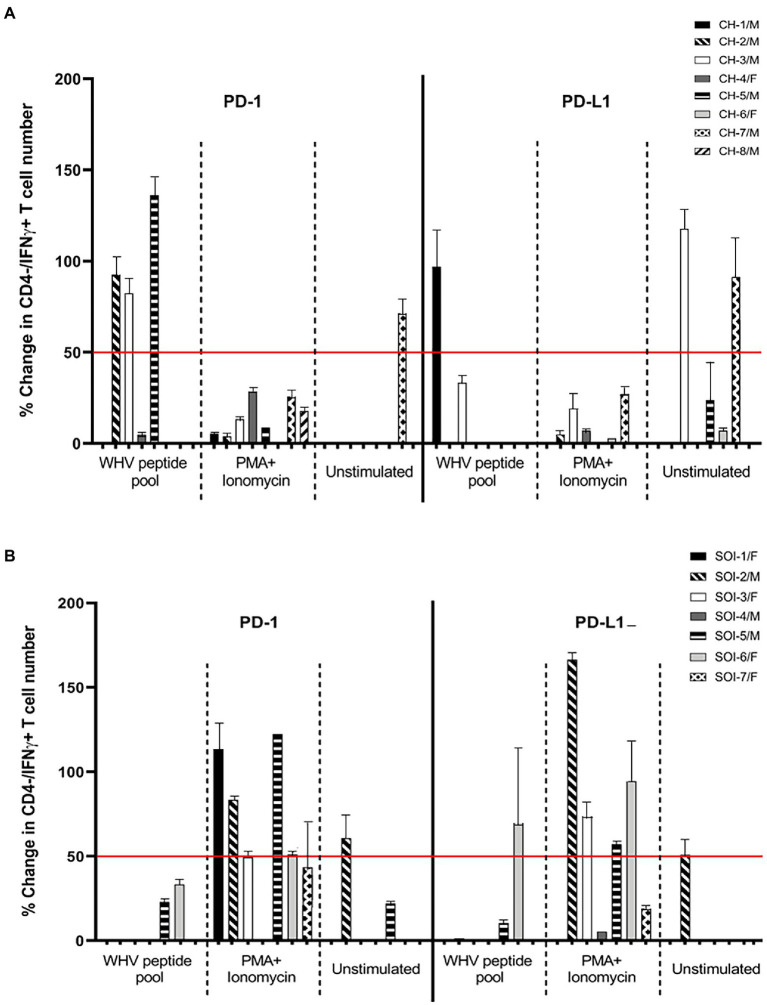
Effect of PD-1 and PD-L1 inhibition on WHV-specific, global and bystander activation of cytotoxic T cells in chronic hepatitis and secondary occult WHV infection continuing after resolution of acute hepatitis. Percentage changes in T cell activation were determined by enumeration of CD3+/CD4–/IFNγ+ T cells after *ex vivo* treatment with anti-PD-1 or anti-PD-L1 and stimulation with WHV peptide pool or PMA/ionomycin or after treatment with the antibodies alone (unstimulated). **(A)** Woodchucks with chronic WHV hepatitis (CH). **(B)** Animals with secondary occult infection (SOI) continuing after self-limited acute hepatitis. Bars represent means with value ranges from usually 2–3 evaluations of PBMC from individual animals. For calculation of the cell number percentage change see “Materials and methods.” Horizontal line at the 50% change is a demarcation between relatively high and relatively low values of the percentage changes.

In this part of the study, PBMC from eight animals with CH and 7 with SOI that continued after self-resolved episode of AH (Table 1) were treated with anti-PD-1 and anti-PD-L1 to determine the Abs capacity to augment WHV-specific and global T cell responses. The outcome of the interference was evaluated by enumeration of CD3+/CD4-/IFNɣ+ T cells, as surrogate of activated CTLs, after stimulation with the total WHV peptide pool or PMA/ionomycin (Figure 5). As controls, cells from the same animals treated with mAb Ig isotype and stimulated with WHV peptides or PMA/ionomycin were used. The data showed a higher level (i.e., change in IFNγ+ T cell number by ≥ 50% comparing to control cells) of WHV-specific T cell activation in 3/8 woodchucks with CH (animals CH-2/M, CH-3/M, and CH-5/M) after treatment with anti-PD-1 and in one (CH-1/M) after exposure to anti-PD-L1 (Figure 5A). One more animal (CH-4/F) demonstrated a small increase (~5%) in WHV-specific T cell activation after PD-1 blockage and another (CH-3/M) a moderate increase (~33%) after treatment with anti-PD-L1. Further, WHV-specific T cell activation greater than 50% was found in one (SOI-6/F) of 7 animals with SOI after exposure to anti-PD-L1 (Figure 5B). The cells of two other animals with SOI (SOI-5/F and SOI-6/F) and another (SOI-5/F) showed activation below 50% after treatment with anti-PD-1 or anti-PD-L1, respectively (Figure 5B). Taken together, the results implied that anti-PD-1 tended be more effective than anti-PD-L1 in activation of WHV-specific CTLs in PBMC from animals with CH, i.e., 3/8 animals with ≥ 50% and 1/8 with < 50% cells activated giving in total 4/8 after anti-PD-1 treatment (range from 0 to 141.2% ± SEM 23.4) versus 1/8 with ≥ 50% and 1/8 with < 50% cells activated resulting in the total of 2/8 animals after treatment with anti-PD-L1 (range from 0 to 114.3% ± SEM 4.5); however statistically significant difference was not achieved (*p* = 0.41). It was also apparent that anti-PD-1 activated WHV-specific CTLs less frequently and to lower levels in PBMC from animals with SOI (*n* = 2; range from 0 to 36.0% ± SEM 1.7) than with in those from CH, but a difference was not statistically significant (*p* = 0.34; Figures 5A,B). Interestingly, activation of WHV-specific CTLs by anti-PD-1 or anti-PD-L1 was predominantly detected in animals with CH infected with WHV shortly after birth. Thus, the activation was seen in 3/4 animals after PD-1 blockage (i.e., CH-2/M, CH-3/M and CH-4/F) and in 2/4 after PD-L1 blockage (i.e., CH-1/M and CH-3/M; Figure 5A). In contrast, T cells from only one (CH-5/M) of the four animals which developed CH after laboratory infection in adulthood showed activation of WHV-specific CTLs after PD-1 and none after PD-L1 blockage (Figure 5A). It should be noted that the observed outcomes of interference with PD-1 or PD-L1 were not related to biochemical severity of CH or serum levels of WHV DNA and WHsAg evaluated at the time of PBMC collection (Table 1 and data not shown). However, activation of WHV-specific CTLs by either anti-PD-1 or anti-PD-L1 was found in animals with a long duration of CH (> 2 years). Thus, mostly in animals with infection acquired during the neonatal period (Figure 5A; Table 1). Only CH-5/M infected as adult demonstrated high numbers of IFNγ+ CTLs after PD-1 blockage. Interestingly, this animal also had CH lasting for 2 years (Figure 5A; Table 1). Other animals infected as adults displayed CH for not longer than 14 mo before collection of PBMC for this study (Table 1) and, as indicated, their T cells did not respond to PD-1 or PD-L1 blockage. This may suggest that T cell dysfunction and/or exhaustion is progressing during CH and that the cells have to achieve certain level of incapacitation before become prone to activation *via* the PD-1/PD-L1 pathway inhibitors.

In contrast to WHV-specific response, PBMC from the majority of animals with CH and SOI displayed CTLs activation after blockage of PD-1 or PD-L1 and stimulation with PMA/ionomycin. Overall, the cells from woodchucks with SOI responded more robustly than these from CH. Thus, activation equal or exceeding 50% over control cell numbers was found in 6/7 animals after exposure to anti-PD-1 (i.e., SOI-1/F, SOI-2/M, SOI-3/F, SOI-5/M, SOI-6/F and SOI-7F) and in 4/7 after treatment with anti-PD-L1 (i.e., SOI-2/M, SOI-3/F, SOI-5/M and SOI-6/F), while treatment with anti-PD-L1 increased CTL numbers by ~20% in PBMC from SOI-7/F (Figure 5B). Contrastingly, none of the animals with CH showed similarly high level of CTL activation after treatment with anti-PD-1 or anti-PD-L1 (Figure 5A). Taken together, the mean numbers of activated CTLs after stimulation with PMA/ionomycin in the presence of anti-PD-1 were significantly greater (*p* = 0.04) for animals with SOI (*n* = 6; range from 0 to 127.3% ± SEM 3.6) than for those with CH (*n* = 7; range from 0 to 32.6% ± SEM 6.4). Treatment with anti-PD-L1, although it appeared to be as effective and gave greater augmentation in IFNγ+ CTL numbers for animals with SOI (*n* = 6; range from 0 to 168.6 ± SEM 4.7) than for animals with CH (*n* = 5; range from 0 to 29.0 ± SEM 2.0), did not result in a statistically significant difference (*p* = 0.07). In contrast to frequent activation of WHV-specific CTLs in animals with long-term CH in response to PD-1 or PD-L1 inhibition, there was no similar association seen after stimulation with PMA/ionomycin.

In addition, activation of T cells was occasionally seen when PBMC from woodchucks with CH or SOI were treated with anti-PD-1 or anti-PD-L1 in the absence of stimulation with either WHV peptides or PMA/ionomycin. This bystander reactivity appeared to be unrelated to the CTL response to WHV peptides or mitogen and was most prominent when PBMC from animals with CH were treated with anti-PD-L1. Among 8 animals in this group, PBMC from 4 animals demonstrated reactivation (range from 0 to 121.4% ± SEM 5.6; Figure 5A). By comparison, this type of T cell activation was found in 2 animals with SOI (range from 0 to 60.7% ± SEM 2.4; Figure 5B).

## Discussion

To expand availability of antibodies for studies on the immune system and its responses in the woodchuck model of hepatitis B and HCC, we embarked on a systematic search and evaluation of commercial Abs recognizing the main types of woodchuck immune cells and functionally important molecules on woodchuck T lymphocytes. Our approach included search and independent determination of woodchuck cDNA sequences and comparison of the deduced amino acid sequences to protein sequences from other species, as well as a review of literature to identify previously utilized cross-reactive antibodies of our interest. The identified regions of homology in the amino acid sequences provided a foundation to uncover potentially cross-reactive Abs in the situation when the antigenic epitopes recognized by these Abs were known. Other factors were also considered in the selection of Abs as outlined in Materials and methods. This foundational analysis resulted in 65 Abs being chosen for investigation (see Table 2). Using flow cytometry approaches, woodchuck PBMC as targets, and a range of control staining, at least one antibody and as many as 4 for almost all woodchuck molecules of interest were identified. The exception was for an antibody against woodchuck CD8 which, irrespective of extensive searching and testing of several candidates, did not result in the identification of this immune cell subtype, particularly when the Ab candidates were tested in combinations with other cross-reactive Abs by multiparametric flow cytometry. Otherwise, 23 Abs recognizing woodchuck T cells (CD3, CD4, and Treg), NK/NKT cells and B cells, as well as T cell-associated PD-1, PD-L1, CTLA-4 and TIM-3 molecules, CD25 and CD69 markers of T cell activation, and IFNγ were either identified or their cross-reactivity with equivalent woodchuck antigenic specificity confirmed (see Table 2). Availability of these Abs should expand opportunities to investigate immunological events in the course of hepadnaviral infection and different stages of hepatitis, and during progression to HCC in the woodchuck-WHV infection model. They also should assist in determining mechanisms of action and in assessing efficacy of novel therapies against HBV and its long-term pathological consequences.

Following confirmation of the cross-reactivity and the blocking competence of Abs against PD-1 and PD-L1, their capacity to activate woodchuck T cells with phenotypic characteristics of CTLs was examined using PBMC from WHV-infected animals. As the results showed, PBMC from woodchucks with CH displayed a highly variable degree of activation of WHV-specific T cells in response to treatment with anti-PD-1. This activation ranged from a high level (i.e., ≥ 50% increase in a number of CD3+/CD4-/IFNγ+ T cells) observed in 3/8 animals to a very low level (~ 5%) in 1/8 and was not measurable in the remaining four (see Figure 5A). In contrast, treatment with anti-PD-L1 caused a high augmentation (≥ 50%) in WHV-specific T cells from one animal and a low level (< 50%) from another, while no effect was seen when PBMC originated from the remaining 6 woodchucks (see Figure 5A). The outcomes regarding anti-PD-L1 treatment roughly resembled these reported for woodchucks with WHV hepatitis in which blockage of the PD-1/PD-L1 pathway was attempted either *in vitro* or *in vivo* with anti-PD-L1 alone, i.e., without co-treatment with an anti-HBV agent or immunization with WHV antigens (Zhang et al., 2011; Liu et al., 2014; Balsitis et al., 2018). These data showed that PBMC from none or a small number of animals responded by activation of WHV-specific T cells as identified by CD107a degranulation assay (Zhang et al., 2011; Liu et al., 2014) or as T cells with the CD3+/CD4-phenotype (Balsitis et al., 2018).

In our study, anti-PD-1 triggered WHV-specific CD3+/CD4-/IFNγ+ T cells more often and to higher levels than anti-PD-L1. For clarity, the effect of anti-PD-L1, but not anti-PD-1, was previously examined in woodchucks with CH (Liu et al., 2014; Balsitis et al., 2018). Although outcomes of interference with the PD-1/PD-L1 pathway may differ in *in vivo* and *in vitro* conditions, the current results suggest that anti-PD-1 either alone or in combination with anti-PD-L1 might activate virus-specific CTLs more robustly and in a larger number of subjects than monotherapy with anti-PD-L1.

In one of the previous studies examining the *in vivo* effect of woodchuck-specific anti-PD-L1, it was noticed that WHV-specific CTLs, defined as CD3+/CD4-T cells, resisted *ex vivo* activation by WHV peptides when PBMC from animals with CH acquired after experimental WHV infection in the early neonatal period were investigated (Balsitis et al., 2018). This contrasted with woodchucks which developed CH following WHV infection in wild since PBMC from some of these animals showed augmentation in WHV-specific CTL response (Balsitis et al., 2018). We also observed a difference in the frequency and the level of activation of WHV-specific CTLs, defined as CD3+/CD4–/IFNγ+ T cells, depending upon how CH was acquired. Thus, anti-PD-1 mAb activated WHV-specific CTLs more often when PBMC originated from CH established after neonatal infection (i.e., 2/4 animals with ≥ 50% and 1/4 with < 50% cells activated) than from animals experimentally infected with WHV as adults (i.e., 1/4 with ≥ 50% cells activated; see Figure 5A). A similar trend was seen after treatment with anti-PD-L1 as PBMC from none of 4 animals with CH acquired after WHV infection in adulthood showed WHV-specific CTL response, while two of the four animals with CH after neonatal infection demonstrated activation of these cells in response to stimulation with WHV peptides, although one responded weakly (< 50% cells activated). These differences however did not achieve statistical significance which was likely due to small numbers of animals with CH in each group. It should be noted that the animals with CH acquired after neonatal infection examined in our study and in the study mentioned above (Balsitis et al., 2018) were purchased from the same supplier (Northeastern Wildlife) and infected with WHV following the same protocol, as indicated in “Materials and methods.” These data emphasize highly individualized outcomes of interference with the PD-1/PD-L1 pathway regarding activation of virus-specific CTLs in chronic hepadnaviral infection. They suggest that detailed recognition of a history of infection, including circumstances of virus acquisition, longevity of chronic hepatitis, the host’s global T cell immune responses, and properties of the antibodies used might be required to predict with greater confidence consequences of the PD-1/PD-L1 blockage in a given individual or animal. This may require *ex vivo* pretesting of the antibody therapeutic effect by using PBMC from an individual in whom the therapy is intended.

WHV-specific T cells with characteristics of activated CTLs were also examined in PBMC of animals with SOI after their exposure to anti-PD-1 or anti-PD-L1. This evaluation was done at 9 to 14 months after serum WHsAg became undetectable, the levels of circulating WHV DNA subsided below 100–200 vge/mL, and biochemical markers of liver injury have normalized. Treatment with mAb against PD-1 or PD-L1 activated to a low level (< 50%) WHV-specific CTLs in PBMC from 2 of 7 animals after treatment with each antibody (see Figure 5B). Notably, WHV-specific activation of CTLs was found in PBMC from the same two animals with SOI after exposure to anti-PD-1 or anti-PD-L1. These data indicated that activation of WHV-specific CTLs could occur months after clinical termination of a symptomatic disease in the context of low-level viral persistence. This finding might be of potential pathogenic as well as therapeutic importance. It is now acknowledged that although the host’s immune system can significantly diminish severity or even terminate hepatitis, it does not mount sterilizing immunity able to eliminate HBV or WHV completely (Michalak et al., 1994; Rehermann et al., 1996; Michalak et al., 1999; Mulrooney-Cousins et al., 2014; Raimondo et al., 2019; Michalak, 2020; Coffin et al., 2021). It is also accepted that there are important pathological and epidemiological consequences of persistent silent HBV and WHV carriage (Michalak et al., 1999; Mulrooney-Cousins et al., 2014; Raimondo et al., 2019; Mak et al., 2020). They include reactivation of infection that may result in severe life-threatening hepatitis, development of HCC, and a risk of virus transmission *via* blood and organ donations compromised by the presence of low levels of virus undetectable by customary testing. From this perspective, augmenting functionality of HBV-specific CTLs, which are the central part of anti-viral immune response, could enhance clearance of residual virus and delay life-threatening outcomes of persistent OBI. This might be particularly effective in combination with agents directly targeting HBV replication. By extension, our findings imply that anti-PD-1/anti-PD-L1 therapy may activate HBV-specific, as well as global and bystander, CTLs in patients with OBI and reactivate hepatitis B. In this context, it is now recognized that CHB patients subjected to therapy with immune check inhibitors targeting PD-1/PD-L1 or CTLA-4 are at a risk of reactivation of HBV infection (He et al., 2021; Ding et al., 2022). This risk is lower in individuals who clinically recovered from an episode of symptomatic HBV infection that is followed by persistent residual virus replication in the liver and at extrahepatic locations (Michalak et al., 1994; Yuki et al., 2003; Raimondo et al., 2019; Michalak, 2020; Coffin et al., 2021). At this stage, a mechanism and aftereffects of OBI reactivation remain uncertain. Nevertheless, there is a possibility that blocking of the PD-1/PD-L1 pathway may disrupt intrahepatic immune homeostasis, augment hepatocyte injury, release silently residing virus, and re-activate inflammation (Knolle and Thimme, 2014; Godbert et al., 2021). Activation of HBV-specific CTLs and CTLs directed against hepatocyte constituents (e.g., autoantigens), which may occur among global and bystander T cell populations, may hypothetically also trigger damage of hepatocytes infected at low levels or those prone to autoimmune-mediated injury (Michalak, 2020). Jointly, precautions should be taken to either exclude or treat with antiviral prophylaxis patients with occult HBV infection in whose administration of inhibitors blocking the PD-1/PD-L1 pathway is considered.

It also was of our interest to assess the effect of blockage of the PD-1/PD-L1 pathway on global activation of CTLs. In this regard, PBMC from animals with CH and SOI were treated with anti-PD1 or anti-PD-L1 when stimulated with PMA/ionomycin and examined for the increase in numbers of IFNγ-positive CD3+/CD4-T cells. The data showed that both anti-PD-1 and anti-PD-L1 activated T cells in PBMC obtained from the majority of animals with CH (i.e., 7/8 treated with anti-PD-1 and 5/8 treated with anti-PD-L1; increases in cells numbers < 50%) and SOI (i.e., 6/7 treated with anti-PD-1 and 6/7 treated with anti-PD-L1; increases in cells numbers ≥ 50% in 4 animals in each of the treatment group; see Figure 5). Overall, generalized (global) activation of CTLs was significantly greater in PBMC from SOI than those from CH after treatment with anti-PD-1 (*p* < 0.05) and also was evident after treatment with anti-PD-L1 although the mean values of increase in the cell numbers did not achieve statistically significant difference. Nevertheless, this clearly showed that CTLs from animals with CH were much less prone to global activation with otherwise very potent T cell stimulator than the cells from animals with SOI. Consequently, this suggested that CTL response to other infectious agents and non-viral antigenic stimuli might be weakened during chronic hepadnaviral hepatitis. This was somewhat unexpected because it has been generally accepted that the decline in the T cell responses in CHB is HBV specific. However, a recent study on the T cell effector and regulatory responses in CHB showed that the T cells’ dysfunction expands beyond the reduced or absent response to HBV and included defective responsiveness to influenza virus and bacterial lipopolysaccharide, another potent global immune cell activator (Park et al., 2016). In the same study, it was also unveiled that there are no distinctive features in T cell functions that can differentiate clinical phenotypes of CHB. In this context, the transiently deficient anti-viral T cell response to influenza virus, Epstein–Barr virus and cytomegalovirus was also reported during AHB in the setting of augmented serum IL-10 and arginase levels (Sandalova et al., 2012). Taken together, our data are consistent with the notion that CH caused by hepadnaviral infection coincides with suppressed both virus-specific and global T cell responses. At this stage, the available data are sparse and further investigations are justified to establish to what degree this global T cell dysfunction modifies natural history and outcomes of immune-mediated and malignant diseases coexisting with CHB. Our results also support the view that HBV-triggered suppression of virus-specific and global T cell responses can be restored to a variable degree, at least in some individuals and animals, when treatment with Abs inhibiting the PD-1/PD-L1 pathway is applied.

Finally, the possibility of bystander activation of CTLs was assessed by treating PBMC with anti-PD-1 or anti-PD-L1 in the absence of WHV-specific or mitogenic stimulation. Increased numbers of CD3+/CD4–/IFNγ+ T cells were sporadically found when PBMC were exposed to either anti-PD-1 or anti-PD-L1. The exception were PBMCs from CH treated with anti-PD-L1 which displayed a variable degree of CTL activation in PBMC from half of the animals investigated (see Figure 5). These increases could be an indication that CD3+/CD4–/IFNγ+ T cells with reactivity towards antigenic specificities other than those triggered by WHV or PMA/ionomycin became activated. This could be possible since cells secreting a variety of antigenic specificities or serving as antigen presenting cells were certainly present in the total PBMC population used as the source of CTLs in our *ex vivo* treatment experiments. However, this may have its equivalence in *in vivo* situation during anti-PD-1/anti-PD-L1 therapy and be responsible for side effects negatively affecting patients. In this regard, increased inflammatory responses affecting different organs including liver, gastrointestinal track, skeletal muscle, skin, endocrine system and even causing immune-related encephalitis have been reported in patients treated with immune checkpoint inhibitors, including anti-PD-1 and anti-PD-L1 Abs [e.g., (Hottinger, 2016; De Martin et al., 2018; Seidel et al., 2018; Ramos-Casals et al., 2020)]. A more profound understanding of the bystander activation of T cells during anti-PD-1/anti-PD-L1 treatment and its potential role in the pathogenesis of side effects could be important in order to limit clinical complications of this otherwise promising immunotherapy.

## Data availability statement

The datasets presented in this study can be found in online repositories. The names of the repository/repositories and accession number(s) can be found in the article/Supplementary material.

## Ethics statement

The animal study was reviewed and approved by Institutional Animal Care Committee at Memorial University, St. John’s, NL, Canada accredited by the Canadian Council on Animal Care in Science. The approved protocol identification number was 13-159-M.

## Author contributions

DW and TM conceived and designed the study. CC and LW performed experiments and with TM and DW analyzed the data. CR and JQ determined woodchuck gene sequences and submitted to GenBank. PM-C designed and provided expertise in cytotoxic T cell assay. MS provided essential expert advice on the study execution and publication. TM and LW wrote the manuscript. All authors listed from Faculty of Medicine, Memorial University, St. John’s, NL, Canada were members of the Molecular Virology and Hepatology Research Group at the time of the data collection and analysis, and there is no conflict of interest with their current affiliations. All authors contributed to the article and approved the submitted version.

## Funding

Elli Lilly and Company funded the research and had no role in the study design, data collection, analysis, decision to publish or manuscript preparation. The funder provided support in the form of salaries for authors (CC, LW, CR, JQ, MS, and DW) and research materials, but did not have any additional role in the study or in preparation of the manuscript. Additional funds came from a research pool grant assigned to TM by Memorial University, St. John’s, NL, Canada, to support in part LW salary, selected research materials and animals, and publication of the manuscript.

## Conflict of interest

CR, JQ, and DW were employed by Elli Lilly and Company. MS was employed by Elli Lilly and Company, Lilly Corporate Center.

The remaining authors declare that the research was conducted in the absence of any commercial or financial relationships that could be construed as a potential conflict of interest.

## Publisher’s note

All claims expressed in this article are solely those of the authors and do not necessarily represent those of their affiliated organizations, or those of the publisher, the editors and the reviewers. Any product that may be evaluated in this article, or claim that may be made by its manufacturer, is not guaranteed or endorsed by the publisher.

## Supplementary material

SUPPLEMENTARY FIGURE S1 Flow cytometry gating strategy used for identification of CD3+/CD4–/IFN+ T cells after stimulation with woodchuck hepatitis virus (WHV) peptides or mitogens and for evaluation of blocking with anti-PD-1 or anti-PD-L1 antibody. In the first step, doublet-cell signal exclusion was performed by plotting forward scatter (FSC)-width against FSC-height (resulting in ~ 95% single cells). This was followed by gating of single cells on FSC-height against side scatter (SSC)-height for debris exclusion (resulting in 90–95% pure cells). The cells were then plotted on FSC-area against Zombie Aqua stained-area to differentiate between live and dead cells (resulting in 95–99% viable cells). Subsequently, live cells were gated on CD3+ cells. To identify activated CTL population, cells were gated as CD4–/IFNɣ+ from CD3+ cells.

SUPPLEMENTARY FIGURE S2 Detection of woodchuck CD3+/IFNγ+ T cells after stimulation of PBMC with PMA/ionomycin or concavalin A. PBMC from a healthy woodchuck **(A)** and an animal with chronic WHV hepatitis **(B)** were treated with PMA/ionomycin or concanavalin A (ConA) or left unstimulated (untreated), and then stained with anti-CD3 alone or with anti-CD3 and anti-IFNγ antibodies, as described in Materials and methods. Total T cell population was gated by SSC and FSC plotting (columns on left). This was followed by the cells quadrant gating for CD3+/IFNɣ+ cells stained with anti-CD3 alone (unstained; columns in middle) or double-stained with anti-CD3 and anti-IFNɣ (IFN-ɣ; columns on right).
