# Supplementary figures and images for: Identification of antibodies cross-reactive with woodchuck immune cells and activation of virus-specific and global cytotoxic T cell responses by anti-PD-1 and anti-PD-L1 in experimental chronic hepatitis B and persistent occult hepadnaviral infection
Source: Front Microbiol. 2022 Dec 6;13:1011070. doi: 10.3389/fmicb.2022.1011070 (PMC9764628; doi:10.3389/fmicb.2022.1011070)

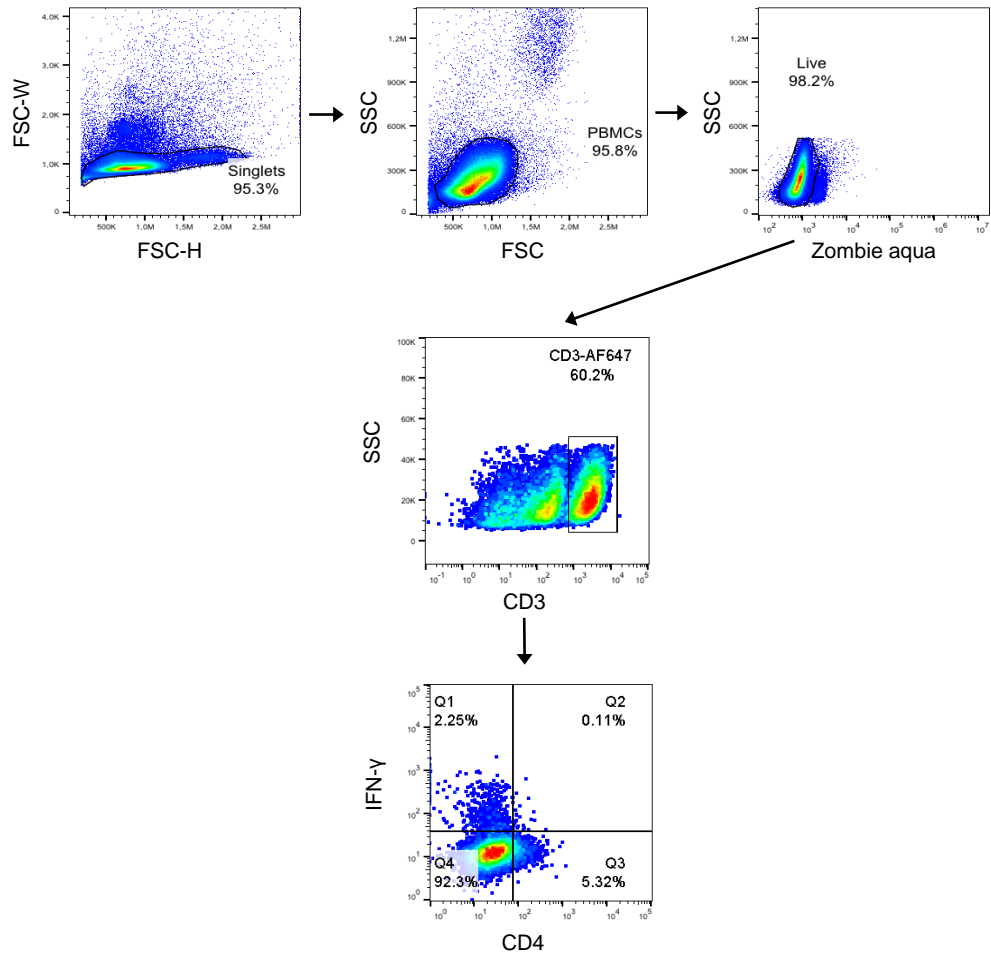

Supplemental Figure 1

Supplement: SUPPLEMENTARY FIGURE S1 — Flow cytometry gating strategy used for identification of CD3+/CD4–/IFN+ T cells after stimulation with woodchuck hepatitis virus (WHV) peptides or mitogens and for evaluation of blocking with anti-PD-1 or anti-PD-L1 antibody. In the first step, doublet-cell signal exclusion was performed by plotting forward scatter (FSC)-width against FSC-height (resulting in ~ 95% single cells). This was followed by gating of single cells on FSC-height against side scatter (SSC)-height for debris exclusion (resulting in 90–95% pure cells). The cells were then plotted on FSC-area against Zombie Aqua stained-area to differentiate between live and dead cells (resulting in 95–99% viable cells). Subsequently, live cells were gated on CD3+ cells. To identify activated CTL population, cells were gated as CD4–/IFNɣ+ from CD3+ cells. [file Data_Sheet_1.PDF]

**A**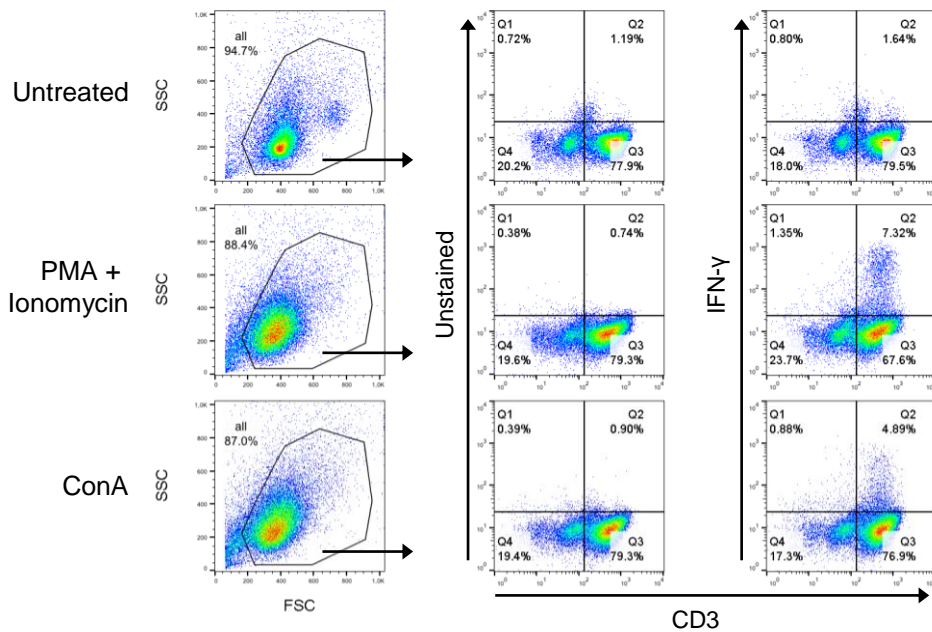**B**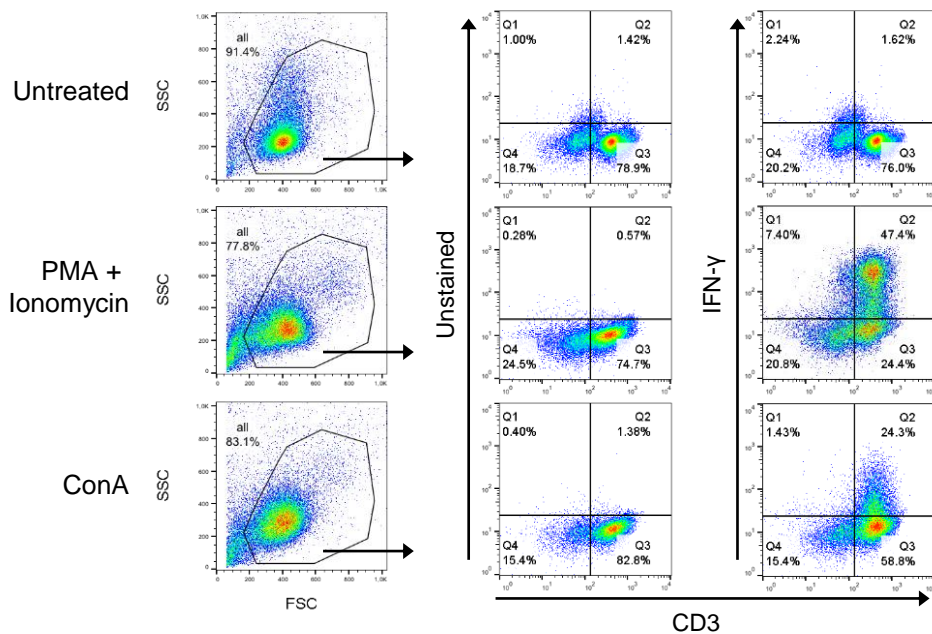

Supplemental Figure 2

Supplement: SUPPLEMENTARY FIGURE S2 — Detection of woodchuck CD3+/IFNγ+ T cells after stimulation of PBMC with PMA/ionomycin or concavalin A. PBMC from a healthy woodchuck (A) and an animal with chronic WHV hepatitis (B) were treated with PMA/ionomycin or concanavalin A (ConA) or left unstimulated (untreated), and then stained with anti-CD3 alone or with anti-CD3 and anti-IFNγ antibodies, as described in Materials and methods. Total T cell population was gated by SSC and FSC plotting (columns on left). This was followed by the cells quadrant gating for CD3+/IFNɣ+ cells stained with anti-CD3 alone (unstained; columns in middle) or double-stained with anti-CD3 and anti-IFNɣ (IFN-ɣ; columns on right). [file Data_Sheet_2.PDF]
